# Probing the Role of Medication, DBS Electrode Position, and Antidromic Activation on Impulsivity Using a Computational Model of Basal Ganglia

**DOI:** 10.3389/fnhum.2016.00450

**Published:** 2016-09-12

**Authors:** Alekhya Mandali, V. Srinivasa Chakravarthy

**Affiliations:** Computational Neuroscience Lab, Department of Biotechnology, Indian Institute of Technology MadrasChennai, India

**Keywords:** deep brain stimulation, parkinson's disease, subthalamic nucleus, impulsivity, electrode position, spiking neuron model, reinforcement learning

## Abstract

Everyday, we encounter situations where available choices are nearly equally rewarding (high conflict) calling for some tough decision making. Experimental recordings showed that the activity of Sub Thalamic Nucleus (STN) increases during such situations providing the extra time needed to make the right decision, teasing apart the most rewarding choice from the runner up closely trailing behind. This prolonged deliberation necessary for decision making under high conflict was absent in Parkinson's disease (PD) patients who underwent Deep Brain Stimulation (DBS) surgery of STN. In an attempt to understand the underlying cause of such adverse response, we built a 2D spiking network model (50 × 50 lattice) of Basal ganglia incorporating the key nuclei. Using the model we studied the Probabilistic learning task (PLT) in untreated, treated (L-Dopa and Dopamine Agonist) and STN-DBS PD conditions. Based on the experimental observation that dopaminergic activity is analogous to temporal difference (TD) and induces cortico-striatal plasticity, we introduced learning in the cortico-striatal weights. The results show that healthy and untreated conditions of PD model were able to more or less equally select (avoid) the rewarding (punitive) choice, a behavior that was absent in treated PD condition. The time taken to select a choice in high conflict trials was high in normal condition, which is in agreement with experimental results. The treated PD (Dopamine Agonist) patients made impulsive decisions (small reaction time) which in turn led to poor performance. The underlying cause of the observed impulsivity in DBS patients was studied in the model by (1) varying the electrode position within STN, (2) causing antidromic activation of GPe neurons. The effect of electrode position on reaction time was analyzed by studying the activity of STN neurons where, a decrease in STN neural activity was observed for certain electrode positions. We also observed that a higher antidromic activation of GPe neurons does not impact the learning ability but decreases reaction time as reported in DBS patients. These results suggest a probable role of electrode and antidromic activation in modulating the STN activity and eventually affecting the patient's performance on PLT.

## Introduction

Parkinson's disease (PD) is a neurodegenerative disorder known to be caused due to the death of dopaminergic neurons in the mid-brain structure called Substantia Nigra pars compacta (SNc) (Obeso et al., 2008) of Basal Ganglia (BG). Apart from the visible motor symptoms such as bradykinesia, rigidity and tremor (Xia and Mao, 2012), cognitive functions of PD patients are also affected (Lees and Smith, 1983; Levin and Katzen, 1994). As an initial treatment, pharmacological medication in the form of dopamine (DA) precursor (L-DOPA) and/or Dopamine agonists (DAA) are prescribed to PD patients (Connolly and Lang, 2014). But it has been observed that the “ON” time (where the medication is effective in relieving the symptoms) decreases as the disease progresses and 80% of the patients develop L-DOPA induced dyskinesias as a side effect (Schrag and Quinn, 2000). Under these circumstances, surgical intervention through Deep Brain Stimulation (DBS) is advised as an alternative treatment wherein an electrode is implanted and external stimulation is given to one or more nuclei of the brain. Though stimulation to the Sub Thalamic Nucleus (STN) of BG is widely followed as the gold standard for PD (Garcia et al., 2005) due to its effectiveness in alleviating the motor symptoms, various experimental studies show a controversial effect of DBS on cognition (Jahanshahi et al., 2000) particularly on impulsivity (Frank et al., 2007; Smeding et al., 2009; Brittain et al., 2012).

Among various experimental paradigms used to study the cognitive ability of PD patients, probabilistic learning task (PLT) (Frank et al., 2004, 2007) captures decision-making ability as well as the impulsivity features. PLT tests the learning capability of the performer not only in choosing rewarding choices but also in avoiding punishing ones. Experimental results show that the performance of normals and PD OFF subjects during PLT is similar in terms of choosing rewarding and avoiding punishing choice (Frank et al., 2007). Contrastingly, the results from the same research group showed a bias toward punishment learning, i.e., the PD OFF subjects learnt better to avoid punitive choice than to choose rewarding choice (Frank et al., 2004) during PLT. The performance of PD ON subjects was opposite to that of PD OFF with a preference toward the rewarding choice, which was accounted by the presence of excess DA levels in the striatum due to medication. This excess DA (due to medication) prevents the PD subjects to learn from punishments. Another critical feature captured by PLT is the reaction time (RT). It has been observed that normal subjects take more time when presented with multiple equally rewarding stimuli (high conflict) and are expected to choose one among them (Frank et al., 2007). Frank et al. (2007) hypothesized that STN increases its activity and buys the extra time needed (“holding the horses”) during such situations. This was further shown by Zaghloul et al. (2012), where an increase in STN activity in PD patients during high conflict conditions was observed (Zaghloul et al., 2012). Experiments conducted by Frank et al. (2007) showed that the performance of DBS subjects on PLT was not significantly different in terms of learning ability but showed impulsive behavior in terms of RT.

Various clinical and experimental studies suggest that the stimulation of STN neurons could lead to a decline in cognitive functions of PD patients (Saint-Cyr et al., 2000; Smeding et al., 2006; Temel et al., 2006; Smeding et al., 2009). Stimulation parameters such as electrode position, pulse frequency and current amplitude seem to play a critical role in altering behavior (Hershey et al., 2004). Using a computational model of subjects/patients performing the Iowa Gambling Task, we earlier showed that the performance of the model in PD with DBS condition showed impulsive behavior, which was further dependent on the position of the electrode and amplitude of the stimulating current (Mandali and Chakravarthy, 2015). Using the same computational BG model (Mandali et al., 2015), we now study the effect of DBS parameters on performance in PLT in terms of accuracy and RT. PLT was simulated using reinforcement learning (RL) framework (Sutton and Barto, 1998), where the temporal difference error term (δ) is hypothesized to resemble the phasic DA released by dopaminergic cells in the midbrain (Schultz et al., 1997).

The aim of this study is two-fold, first to show that the spiking BG model is able to replicate the performance of normal, PD OFF, PD ON (L-DOPA) conditions as in experimental studies (Frank et al., 2007) and secondly to hypothesize the effect of DAA, DBS electrode and antidromic activation on learning, impulsivity and behavior.

## Materials and methods

We used the spiking neuron model of the Basal Ganglia (BG) (Mandali et al., 2015) in normals, PD OFF, PD ON (L-DOPA and DAA), and DBS (electrode position and antidromic) conditions to simulate PLT (Frank et al., 2004, 2007). The various performance measures used to validate the model results are also introduced in this section.

### Spiking neuron model of basal ganglia

**Figure 1:**
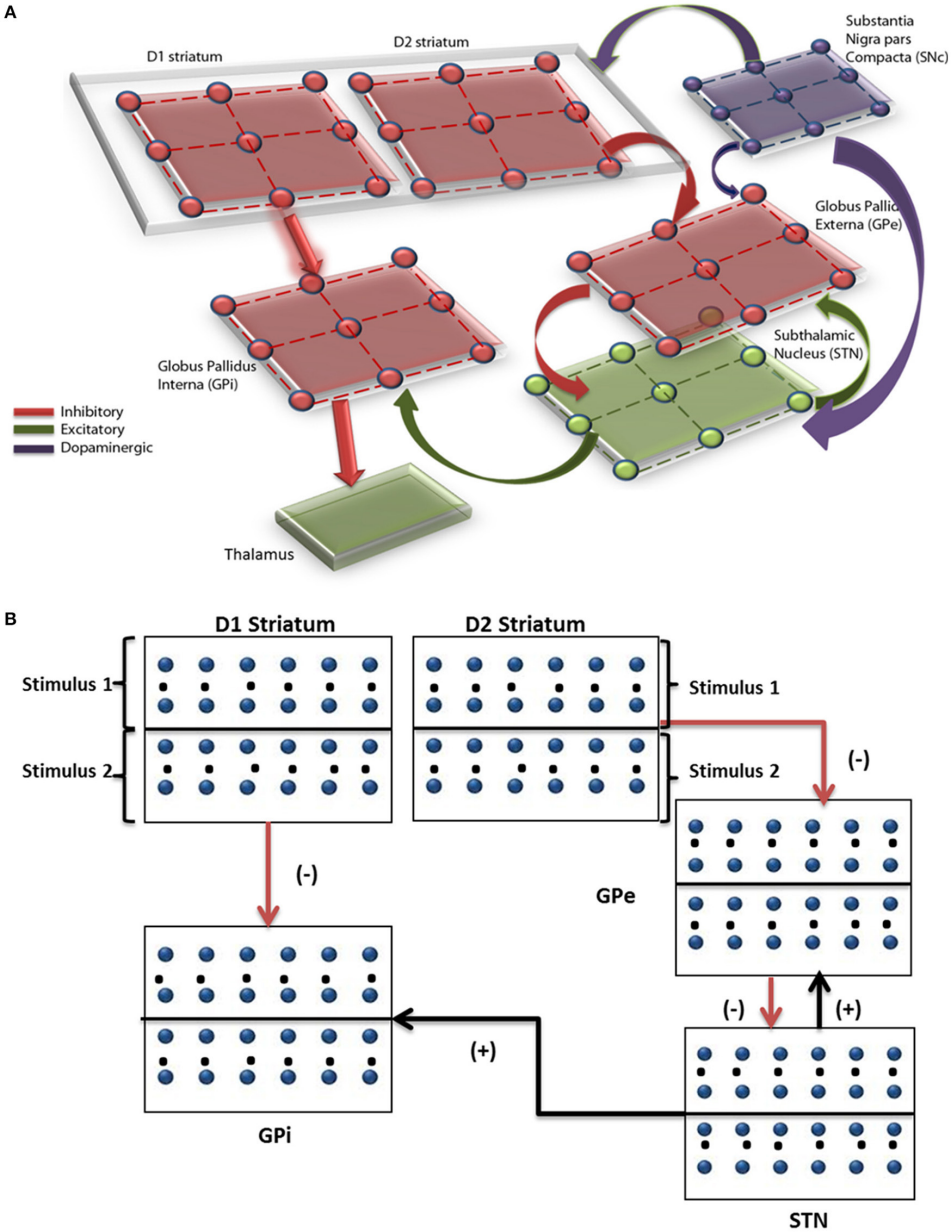
**Pictorial representation of the spiking basal ganglia model with all the key nuclei such as striatum, STN, GPi, GPe, SNc, and thalamus**. **(A)** The synaptic projections were modeled as glutamatergic indicated by green color and GABAergic currents in red color. **(B)** Shows a graphical picture of how input stimuli were presented to the model.

The network model of BG (Mandali et al., 2015) (Figure 1) was built using 2-variable Izhikevich spiking neurons (Izhikevich, 2003) where each nucleus was modeled as a 2D array of neurons. Parameters for each of the nuclei [STN, Globus Pallidus externa (GPe), and interna (GPi) were chosen; (Mandali and Chakravarthy, 2015; Mandali et al., 2015)] to resemble their biological counterparts. STN and GPe neurons are bi-directionally connected (Plenz and Kital, 1999) in one-to-one fashion where GPe (STN) projections are inhibitory (excitatory). The striatum which receives input from the cortex (Tritsch and Sabatini, 2012; Silberberg and Bolam, 2015) consists of both D1R-expressing and D2-R expressing medium spiny neurons (MSNs) and was segregated based on the classical anatomical classification of direct and indirect pathways (Gerfen and Surmeier, 2011) and was modeled as Poisson spike trains modulated by DA levels. Each GPi neuron receives both glutamatergic projection from STN and GABAergic projection from D1 MSN. Similarly, each GPe neuron receives GABAergic input from D2 MSN and glutamatergic from STN neuron. The full set of equations related to the Izhikevich spiking neuron model are described in Appendix A and Table A.I (Supplementary Materials). The input from the cortex to STN, also known as the hyper-direct pathway (Nambu, 2015), and the GABAergic projection from GPe to GPi were not included in the model as the functional significance of these connections is not fully understood.

(1) $$\frac{dv_{ij}^{x}}{dt}=0.04{\left(v_{ij}^{x}\right)}^{2}+5v_{ij}^{x}-u_{ij}^{x}+140+I_{ij}^{x}+I_{ij}^{syn}+I_{ij}^{DBS}$$

(2) $$\frac{du_{ij}^{x}}{dt}=a(bv_{ij}^{x}-u_{ij}^{x})$$

(3) $$if\;v_{ij}^{x}\geq v_{peak}\left\{\begin{array}{l} v_{ij}^{x}\leftarrow c\;\\ u_{ij}^{x}\leftarrow u_{ij}^{x}+d \end{array}\right\}$$

where, $v_{ij}^{x}$ = membrane potential, $u_{ij}^{x}$ = membrane recovery variable, $I_{ij}^{Syn}$ = total synaptic current received, $I_{ij}^{x}$ = external current applied to neuron x at location (*i, j*), *v*_*peak*_ = maximum voltage set to neuron (+30 mv) with x is a generalized notation denoting either STN, GPe or GPi neuron and $I_{ij}^{DBS}$ is the stimulation current applied only for STN neurons (defined in the next Section Simulating Various Conditions in the Model).

### Simulating various conditions in the model

The methods and parameters used in the model to simulate the task in normal, PD OFF, PD ON (L-DOPA and DAA), and DBS conditions are explained in this section.

#### Normals

To simulate the normal condition, the direct pathway (D1 MSN to GPi) weight in spiking BG model was kept high (w_Str→D1_ = 4) and the weight from STN to GPi low (w_STN→GPi_ = 1.5). The lateral weights within STN and GPe were kept at *w*_*sg*_ = 0.91 and *w*_*gs*_ = 18 based on the experimental evidence that there is high amount of inhibition from GPe to STN in normal conditions (Wilson and Bevan, 2011). The radius of neighborhood of connectivity in STN (*r*_*s*_ = 1.4) and GPe (*r*_*g*_ = 1.6) which controls the level of synchrony in the nuclei, is chosen such that the STN-GPe system exhibits desynchronized dynamics as observed in the normal healthy condition (Bergman et al., 1998; Wilson and Bevan, 2011).

#### Parkinsonian condition in “OFF” and “ON” state

##### PD OFF state

Bearing in mind that the TD error (δ) is similar to the DA activity (O'doherty et al., 2003; Schultz, 2007; Rolls et al., 2008) and there is loss of DA neurons in PD, we simulated it by clamping the “δ” value to a low limit (δ_lim_ = −0.1) representing a loss of dopaminergic neurons in PD condition (Equation 4).

(4) $$\delta_{\lim}=\min(\delta ,DA_{ceil})$$

where *z* = min(*y, a*)is defined as $\begin{array}{l} z=y\;if\;y<a\\ \;a\;if\;y>a \end{array}$

We also decreased the direct pathway weight (w_*Str*→*D*1_ = 3) which represents a decreased inhibitory output from D1 striatum to GPi and increased STN to GPi weight (w_*STN*→*GPi*_ = 2) representing an increase in the excitatory input from STN. The remaining parameters were not varied.

##### PD ON state

PD “ON” medication clinically involves external intake of dopamine precursors such as L-DOPA. A simple way to simulate oral medication is to add a “δ_med_” term to the δ_lim_ term (Equation 4) (Magdoom et al., 2011; Muralidharan et al., 2013; Mandali and Chakravarthy, 2015).

(5) $$\delta_{\text{new}}=\delta_{\text{lim}}+\delta_{\text{med}}$$

Another class of medication that is prescribed to PD patients is Dopamine Agonists (DAAs), which could be receptor specific. Here we simulated DAA action in such a way that it precisely effects D2 class of receptors, which are also linked to impulsivity (Macmahon and Macphee, 2008). Therefore, in case of DAA, the parameter δ_new_ in Equation (5) will be used to update only D2 cortico-striatal weight (w^D2^, Equation 8) unlike for L-DOPA where both w^D1^ and w^D2^ were updated. All the parameter values of the model are kept the same as in PD OFF state, except the weight parameters which are reverted to normal. This is one among many approaches used to simulate the effect of dopaminergic medication. The medication is added to the model as described in Equation (5) where (δ_med_ = 2) is added to the clamped delta (δ_lim_ = −0.1). The two types of dopaminergic medications (L-DOPA and DAA) differ only in terms of weight update as described in next Section (Simulating PLT Using Spiking BG Model).

#### DBS stimulation

The effect of DBS on the STN neurons was modeled by giving an external stimulation current (Equation 6). The parameters (frequency, pulse duration, and amplitude) of the stimulation current are chosen such that they are comparable to that used in a clinical setting (Garcia et al., 2005). The stimulation current is given to the entire/part of STN module (50 × 50 neurons) in the form of Gaussian distribution (Hauptmann and Tass, 2007; Foutz and McIntyre, 2010; Mandali and Chakravarthy, 2015). The mean of the Gaussian coincides with the lattice position (*i*_*c*_, *j*_*c*_) which is assumed to be the center of the electrode and the extent of the current spread is controlled by the Gaussian width (σ).

(6) $$I_{ij}^{DBS}=A^{*}e^{\frac{-({\left(i-i_{c}\right)}^{2}+{\left(j-j_{c}\right)}^{2})}{\sigma^{2}}}$$

where $I_{ij}^{DBS}$ is the DBS current received by the STN neuron at position (*i, j*), A is the amplitude of the current (pA), σ controls the spread of the current, and (*i*_*c*_, *j*_*c*_) is the mean/center point of the electrode. The effect of electrode position (*i*_*c*_, *j*_*c*_) and stimulation parameters A and σ on STN activity and on decision making behavior is simulated.

All the synaptic weight values are kept similar to that of PD OFF condition and external current (I_*DBS*_) as described in Equation (6) was added to the STN neurons. The current was applied at a frequency of 130 Hz, mostly monophasic mode with pulse duration = 100 μS, the spread of the current σ = 5 and amplitude of the current around 220 pA with the electrode center at the lattice point (25, 25).

##### Electrode position

Experimental results show that change in the electrode position alters behavior (Hershey et al., 2004, 2010) and this can be attributed to the difference in pattern and volume of STN activation due to the electrode position (Miocinovic et al., 2006). Also, the final action or choice selection depends on the activity of GPi neurons which receive weighted input from STN and D1R-expressing MSNs. Bearing these points in mind, we chose three electrode positions where the lattice point indicates the center of the electrode, i.e., Pos 1 in the upper half of the STN nucleus at lattice point (13, 13), Pos 2 with electrode contact center at the lattice point (25, 25), and Pos 3 in the lower half of the STN nucleus at lattice point (38, 38). Each module (StrD1, StrD2, GPe, and STN) in the model is divided into four quadrants corresponding respectively to the four panels in the PLT. This is a modeling assumption that has to be made in the absence of experimental data about how the four action choices might be represented in the basal ganglia nuclei. The electrode position that we study in the model is also described with reference to such representations. Thus, the four quadrants in the modules do not correspond to the well-known basal ganglia loops like sensorimotor, associative, limbic etc.

##### Antidromic activation

Based on theories that stimulation of STN could result in antidromic activation of GPe, GPi, or cortical neurons (Hauptmann and Tass, 2007; Montgomery and Gale, 2008), we studied the effect of antidromic activation of GPe neurons during the task. This effect was modeled by adding a percentage of DBS current (given to STN neurons) directly to GPe neurons. The antidromic activation of GPe neurons in the network was simulated by providing certain percentage of DBS current (Equation 6) to the GPe neurons. For example, 25% antidromic activation would have 25% of the DBS current ($I_{ij}^{DBS}$) added to the membrane potential equation (Equation 1) of GPe neurons and the remaining 75% to STN neurons.

### Probabilistic learning task (PLT)

**Figure 2:**
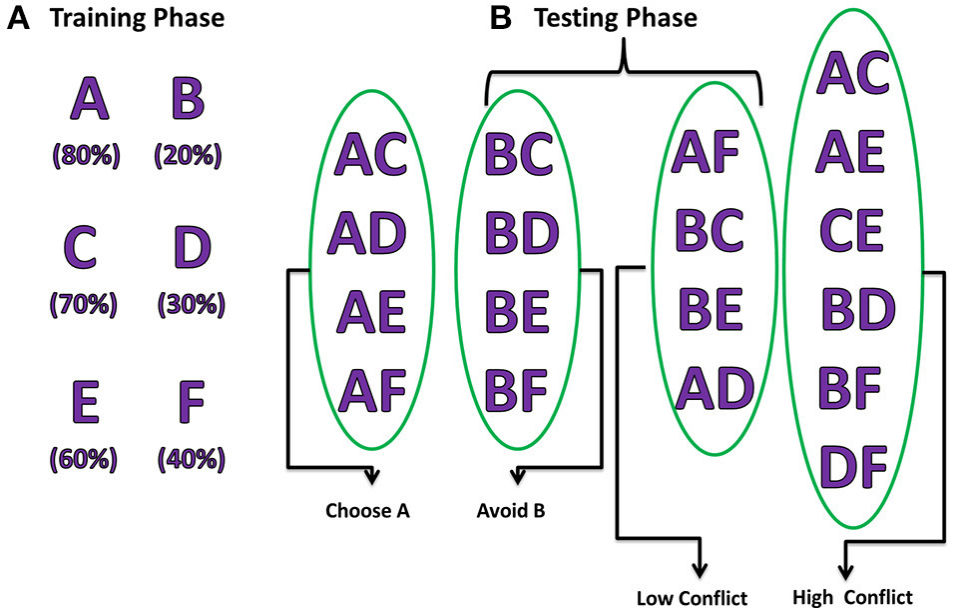
**Pictorial representation of probabilistic learning task (Frank et al., 2007) indicating training and testing stages**. The percentages below each choice in the training phase indicate their corresponding reward probabilities. The testing stage is divided into 2 steps: **(A)** Positive/Negative learning testing the “Choose A” and “Avoid B” (Frank et al., 2007), **(B)** test reaction time during LC and HC conditions.

The experiment consists of two stages, training and testing (Figure 2). During the training stage, the model was presented only with three pairs of stimuli (AB/CD/EF) one at a time in a random fashion. Each of the six choices (A/B/C/D/E/F) was associated with a reward with *a priori* probability. For example, selection of choice “A” leads to reward (= +1) 80% of the time whereas choice “B” leads to a reward only 20% of the time. Similarly, choice “C” (“E”) gives reward with a probability of 70% (60%) and choice “D” (“F”) leads to reward only 30% (40%) of the time and punishment (= −1) for rest of the trials. The model was expected to learn these reward probabilities by the end of training.

During the testing stage, the model was tested with 15 novel combinations (e.g., AC, CE, DE) which were not presented during the training stage. No feedback was provided for the response made after each stimulus. The model was tested for its learning ability based on whether it chose (avoided) a rewarding (punishing) choice from the presented combination pair. For example, if a novel combination of choice “A” with another choice was presented; the model was expected to choose “A” as the probability of obtaining a reward was the highest for “A.” Similarly, when the stimuli with combination of “B” with other choices were presented, the model is expected to avoid selecting “B” as its reward probability was the lowest. Apart from testing for the learning ability, the model was also tested for performance during High conflict (HC) and Low conflict (LC) situations. For example, the stimulus combination “AC” falls under the category of HC as both choice “A” (80%) and “C” (70%) have high reward probabilities but stimulus combination “BC” comes under the category LC as reward probabilities (“B” = 20% and “C” = 70%) are significantly different. The reaction time was measured for each of the conditions (HC/LC).

During this stage, the model was tested for the following conditions:

- Testing accuracy where the model was presented with 15 novel combinations not used during the training phase.
- Choice/Avoidance Accuracy of the model to select choice “A” and avoid choice “B” when presented with all possible novel combinations containing either “A” or “B.”
- Decision making efficiency in term of reaction times during HC and LC situations.

#### Simulating PLT using spiking BG model

During training and testing phase, a pair of choices was presented out of the six choices in each trial, so the input to the BG model was also given as a set of two inputs. Every nucleus in the model was divided equally into two parts which receive the corresponding input. The expected reward probability of the corresponding input was learnt in the cortico-striatal weights. Based on the experimental evidence that striatal neural spiking activity is irregular (Reti, 2015), the input to GPe and GPi (i.e., the output of D2R- and D1R-expressing striatal MSNs) was modeled as Poisson spike trains whose frequency is proportional to the cortico-striatal weight ($w_{i,k}^{D1}$,$w_{i,k}^{D2}$) of the corresponding stimulus pair (i) and trial (k). Since the rate of firing of the striatal neurons was observed to be between 2 and 40 Hz (Kravitz et al., 2010), the cortico-striatal weight of the individual card was normalized to fall in the above range. Since release of DA is known to modulate plasticity (Surmeier et al., 2007) in cortico-striatal connections, in the present model, the temporal difference error term “δ” was used to update the cortico-striatal synapses (Reynolds and Wickens, 2002; Surmeier et al., 2007). The synaptic weights between all other nuclei were not plastic and were changed only depending on the physiological condition.

##### Cortico striatal weight update and temporal difference error

Each choice (A/B/C/D/E/F) was associated with 2 weights ($w_{i,0}^{D1}$,$w_{i,0}^{D2}$) which were initialized with random values selected from a uniform distribution over (0, 1). The two weights represent the cortico-striatal weights of D1R and D2R-expressing striatal MSNs and are trained as,

(7) $$\Delta w_{i,k}^{D1}=\eta \delta_{k}x_{i,k}^{inp}$$

(8) $$\Delta w_{i,k}^{D2}=-\eta \delta_{k}x_{i,k}^{inp}$$

The expected value (V_*k*_) for kth trial, which is expressed in terms of the activity of D1R-expressing MSNs (Chakravarthy et al., 2010; Muralidharan et al., 2013; Mandali and Chakravarthy, 2015; Mandali et al., 2015), is calculated as

(9) $${\text{V}}_{\text{k}}=\sum_{i\;=\;1}^{6}w_{i,k}^{D1}*x_{i,k}^{sel}$$

The gain or reward (Re_*k*_) for kth trial is calculated as

(10) $${\text{Re}}_{\text{k}}=\sum_{i\;=\;1}^{6}r_{i,k}*x_{i,k}^{sel}$$

The error (δ) for kth trial is defined as

(11) $$\delta_{k}={Re}_{k}-V_{k}$$

where,

$w_{i,k}^{D1}$ are the cortico-striatal weights of D1 striatum for ith card in kth trial, $w_{i,k}^{D2}$are the cortico-striatal weights of D2 striatum for ith card for kth trial,

Card “i” represents one of the six cards (A/B/C/D/E/F)

*r*_*i, k*_ is the reward obtained for the selected ith card in kth trial

x^inp^ is the binary input vector representing the choices presented to the model each time, e.g., if the stimulus presented is CD, x^inp^ = [0 0 1 1 0 0] and x^sel^ is the binary vector representing the choice that got selected; if “C” is selected then x^sel^ = [0 0 1 0 0 0]; η (=0.1) is the learning rate of the cortico-striatal synapses of D1 and D2 MSNs; V_k_ is the expected value for the selected card for kth trial.

### Performance measures

In this section, we explain all the performance measures used in this study to quantify and validate the results obtained from the model for all the conditions.

#### Learning

The model was trained for 120 trials [=40 per combination (AB/CD/EF)] and the learning ability of the model was checked during the training stage in terms of training accuracy where the probability of selecting the correct choice was plotted as the training progressed (trials were divided into five equal bins). The performance of the model was compared with the results (Figure 2A from Zaghloul et al., 2012).

#### Testing accuracy and difference in reward expectation (DRE)

##### Difference in reward expectation (DRE)

After training, the *a priori* choice selection probability was calculated based on the number of times the corresponding choice was presented and selected. We then calculated the Difference in Reward Expectation (DRE), which is the difference between the 2 apriori choice probabilities for that particular presented stimulus. DRE captures the amount of conflict between the presented choices, the higher (lower) the DRE for that stimulus the lower (higher) is the conflict. For example, if stimulus “BC” was presented then DRE_BC_, which is the difference between P(B) and P(C), would be low, thereby reducing the probability of choice “B” getting selected.

##### Testing accuracy

Once the training phase is completed, the model was tested by presenting 15 novel combinations. The objective was to calculate the probability with which the first choice in the presented stimulus was selected. For example, if stimulus “AC” was presented for 20 times and choice “A” was selected for 16 times, then the testing accuracy for choice “A” would be 0.8 (=16/20).

The learning ability of a system to select the most rewarding choices while avoiding the punitive ones can be obtained by just evaluating the relationship between DRE and testing accuracy. For example, the testing accuracy (of choice “A”) for the stimulus “AF” (whose DRE > 0) would be expected to be high because the reward probability associated with choice A is also high. So for an optimally trained system, one can expect a linear relationship between testing accuracy and DRE.

#### Choice/avoidance accuracy

This quantity measures the ability of the model to select the most rewarding option “A” and avoid the punitive choice “B” when presented with novel combinations not used during training.

#### Reaction time

The final action selection was done at the level of thalamus which was simulated using the “race model” with mutual inhibition (Bogacz et al., 2006) where an action is selected when temporally integrated neuronal activity of the output neurons crosses a threshold (Frank, 2006; Frank et al., 2007; Humphries et al., 2012).

The dynamics of the thalamic neurons is as follows,

(12) $$\begin{array}{l} \frac{dz_{1}\left(t\right)}{dt}=-z_{1}\left(t\right)+f_{Gpi1}(t)-z_{2}\left(t\right)\\ \frac{dz_{2}\left(t\right)}{dt}=-z_{2}\left(t\right)+f_{Gpi2}(t)-z_{1}\left(t\right) \end{array}$$

(13) $$\begin{array}{l} f_{Gpik}^{\prime }=\frac{1}{(N*N)/k}\overset{T}{\underset{t=1}{\sum }}(\overset{N}{\underset{i=1}{\sum }}\overset{N/k}{\underset{j=1}{\sum }}S_{ij}^{GPik}(t))\\ f_{GPik}=\frac{f_{GPi}^{\max}-f^{\prime }Gpik}{f_{GPi}^{\max}} \end{array}$$

where, *z*_1_(*t*), *z*_2_(*t*) = integrating variable for 1st and 2nd choice, *f*_*GPi*1_(*t*) and *f*_*GPi*2_(*t*) = normalized and reversed average firing frequency of GPi neurons receiving 1st and 2nd choice from striatum, $f_{GPi}^{\max}$ = highest firing rate among the GPi neurons, $S_{ij}^{GPik}$ = neuronal spikes of GPi neurons receiving kth stimulus, N = number of neurons in a single row/column of GPi array (=50), T = duration of simulation.

The first neuron (z_*k*_) among k stimuli to cross the threshold (=0.25) represents the action selected and “t” is the time instant when the action gets selected which is nothing but the RT. All the variables representing neuronal activity are reset immediately after each action selection.

## Results

The simulation study was performed to study various aspects of behavior in Normals, PD OFF, PD ON (L-DOPA and DAA), and DBS conditions. Table 1 presents list of simulation (from the model) and experimental measures for various conditions.

**Table 1:** **Table shows the overview of all the results obtained from the model and compared with experimental results**.

**S.No**	**Performance measure**	**Condition**
1	Learning	Normals^*^
		PD OFF^a^
		PD ON (L-Dopa)^*^
		PD ON (DAA)^*^
		DBS^*^
		DBS (Electrode Position)^*^
		DBS(Antidromic activation)^*^
2	Testing accuracy vs. DRE	Normals^*^
		PD OFF^a^
		PD ON (L-Dopa)^*^
		PD ON (DAA)^*^
		DBS^*^
		DBS (Electrode Position)^*^
		DBS(Antidromic activation)^*^
3	Choice/avoidance accuracy	Normals^b^
		PD OFF^b^
		PD ON (L-Dopa)^b^
		PD ON (DAA)^*^
		DBS^b^
		DBS (Electrode Position)^*^
		DBS(Antidromic activation)^*^
4	Reaction time and impulsivity	Normals^b^
		PD OFF^b^
		PD ON (L-Dopa)^b^
		PD ON (DAA)^*^
		DBS^b^
		DBS (Electrode Position)^*^
		DBS(Antidromic activation)^*^

* *Experimental literature not available for these conditions*.

a *Zaghloul et al., 2012*.

b *Frank et al., 2007*.

### Learning

**Figure 3:**
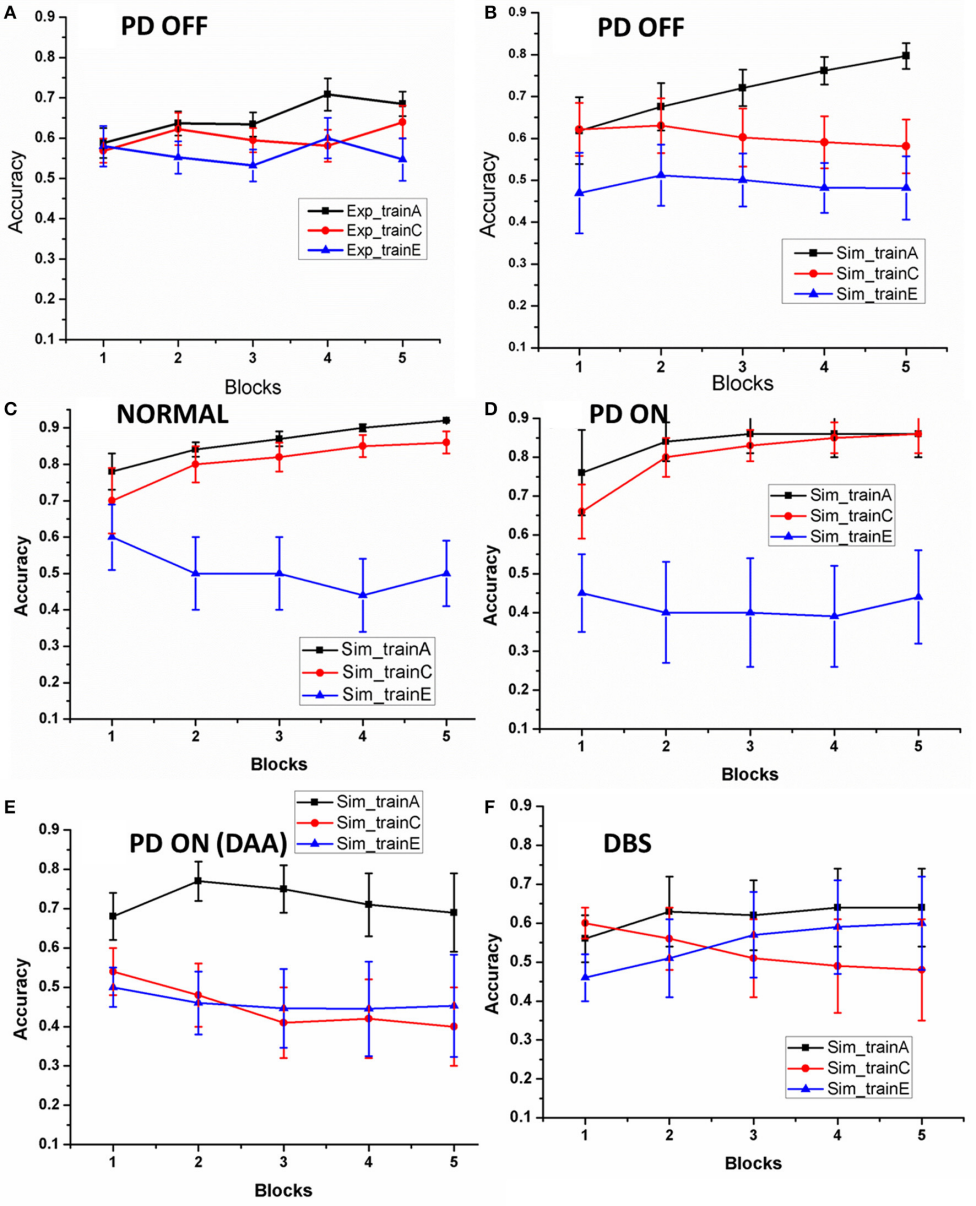
**The accuracy of the model as the training progressed: (A) results redrawn from Zaghloul et al. (2012), (B) performance of the spiking model in PD OFF condition, (C) Normal, (D) PD ON, (E) PD ON (DAA), and (F)DBS**. The x-axis is the progression in training trials which were divided in to five equal blocks. The y-axis indicates the mean accuracy with standard error (SE).

As explained in Section Learning, we evaluated the training ability of the model for Normals, PD OFF, PD ON (L-DOPA and DAA), and DBS conditions. The training ability in PD OFF condition was compared with experimental results (Figure 2A of Zaghloul et al., 2012). As shown in Figure 3, the training accuracy levels (probability of choosing the particular choice during training) for choices “A,” “C,” “E” reach their actual reward probabilities (0.8/0.7/0.6) as the training progresses. Table 2 shows the accuracy levels of choosing “A,” “C,” and “E” for all the above conditions.

**Table 2:** **Shows the training accuracy levels for choosing “A” [P (A)], choosing “C” [P(C)], and choosing “E” [P(E)] in normal, PD OFF, PD ON (L-DOPA and DAA), and DBS conditions**.

**Condition**	**P(A)**	**P(C)**	**P(E)**
Normal	0.92 ± 0.001	0.86 ± 0.03	0.5 ± 0.09
PD OFF	0.79 ± 0.03	0.58 ± 0.06	0.48 ± 0.07
PD ON (L-DOPA)	0.86 ± 0.06	0.85 ± 0.05	0.44 ± 0.12
PD ON (DAA)	0.69 ± 0.1	0.4 ± 0.1	0.45 ± 0.13
DBS	0.64 ± 0.1	0.48 ± 0.13	0.6 ± 0.12

By the end, the training accuracy for “A,” “C,” and “E” in normal, PD OFF and PD ON (L-DOPA) reached their reward probabilities (Figures 3B–D) showing the learning ability of the model in that condition. But PD ON (DAA) and DBS conditions (Figures 3E,F) showed lower training accuracy compared to other conditions.

### Testing accuracy and difference in reward expectation (DRE)

**Figure 4:**
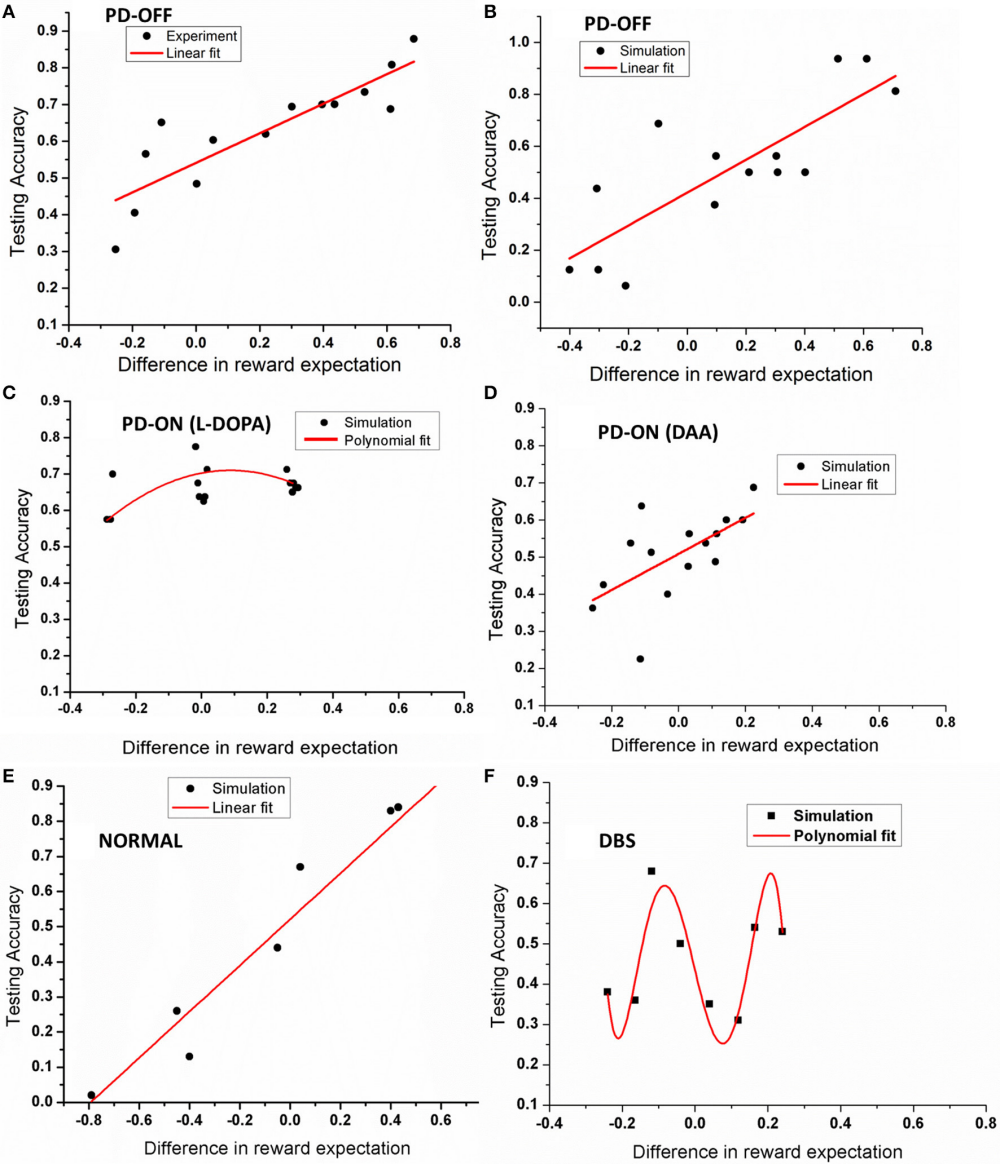
**Shows an example plot of testing accuracy against DRE for (A) PD OFF experiment, (B) PD OFF simulation, (C) PD ON (L-DOPA), (D) PD ON (DAA), (E) Normal, and (F) DBS conditions**. X-axis shows difference in reward expectation (DRE) and Y-axis indicates the performance during testing.

The model was then tested with 15 novel stimuli consisting of all the combinations of the choices (A–F). Linear regression was used to fit the testing accuracy as a function of DRE for most of the above conditions. The results of PD OFF obtained from the model (Figure 4B) were compared with results (Figure 2C) from that of Zaghloul et al. (2012) (Figure 4A). The fit of the regression line for experiment (=0.81) and that obtained from simulation (=0.87). The testing accuracy obtained from experiment and simulation were compared using *t*-test and found not to be significantly different (*p* = 0.16). We also studied the same for PD medication “ON” conditions (both L-DOPA and DAA). As one can observe, the testing probability is confined to top left corner in PD ON (L-DOPA) condition (Figure 4C). The PD-ON (L-DOPA) condition did not show a linear relationship between testing accuracy and DRE. The DRE obtained for all the novel combinations did not cross 0.24 and the testing accuracy is high even in negative DRE conditions (Figure 4D). A good fit was not obtained for the L-DOPA condition, the better polynomial (with order 2) is reported below. This suggests that L-DOPA interferes with training leading to wrong estimation of the reward. We also checked the same for normal condition (Figure 4E) and the results showed a low testing accuracy for low DRE and vice versa with a regression of (=0.94). The testing accuracy for DBS condition (Figure 4F) also showed a better correlation only for a polynomial fit of order 5 (=0.47). Table 3 lists the goodness of fit obtained from the model for testing accuracies and DRE for all the conditions.

**Table 3:** **Table explains the goodness of fit for the simulation results obtained from testing accuracies and DRE for all the conditions**.

**Condition**	**Type of fit**	***R*-value**
Normal	Linear	0.94^*^
PD-OFF	Linear	0.81^*^
PD-ON (L-DOPA)	Polynomial (order = 2)	0.01
PD-ON (DAA)	Linear	0.32^*^
DBS	Polynomial (order = 5)	0.47

* *Indicates the significance level at p = 0.05*.

Apart from studying the relationship between testing accuracy and DRE (in terms of regression fit), we wanted to know if there was fundamental difference in absolute values of the testing accuracies obtained from each of these conditions. So we conducted one way ANOVA and found the testing accuracies to be significantly different at *p* = 0.05 level with [*F*_(2, 40)_ = 3.62, *p* = 0.03].

The training and testing accuracy results from the model show its ability to capture the behavior of PD patients. The testing accuracy results (with DRE) suggest how medication and DBS could affect the decision making ability.

### Choice/avoidance accuracy

**Figure 5:**
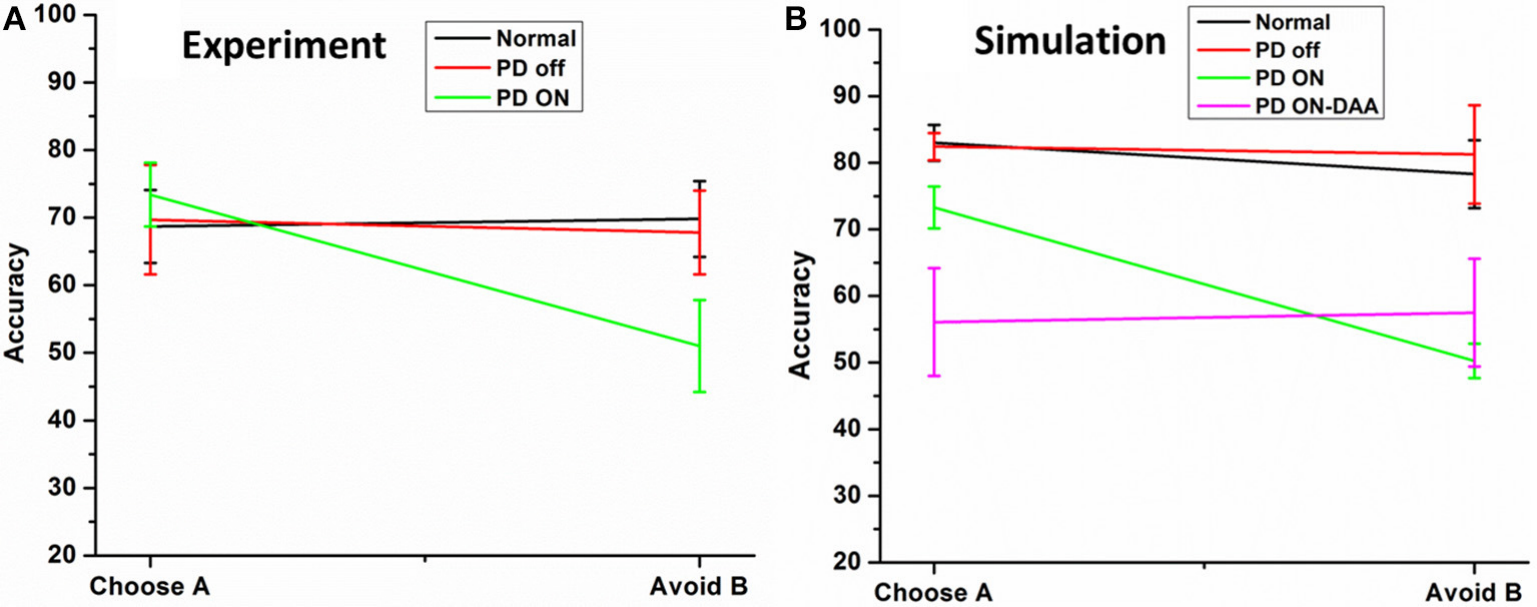
**Shows the testing accuracy of the model in for HC, PD Off, PD ON (L-DOPA and DAA). (A)** Shows the experiment results redrawn from Frank et al. (2007) **(B)** simulation results obtained from the spiking BG model.

**Figure 6:**
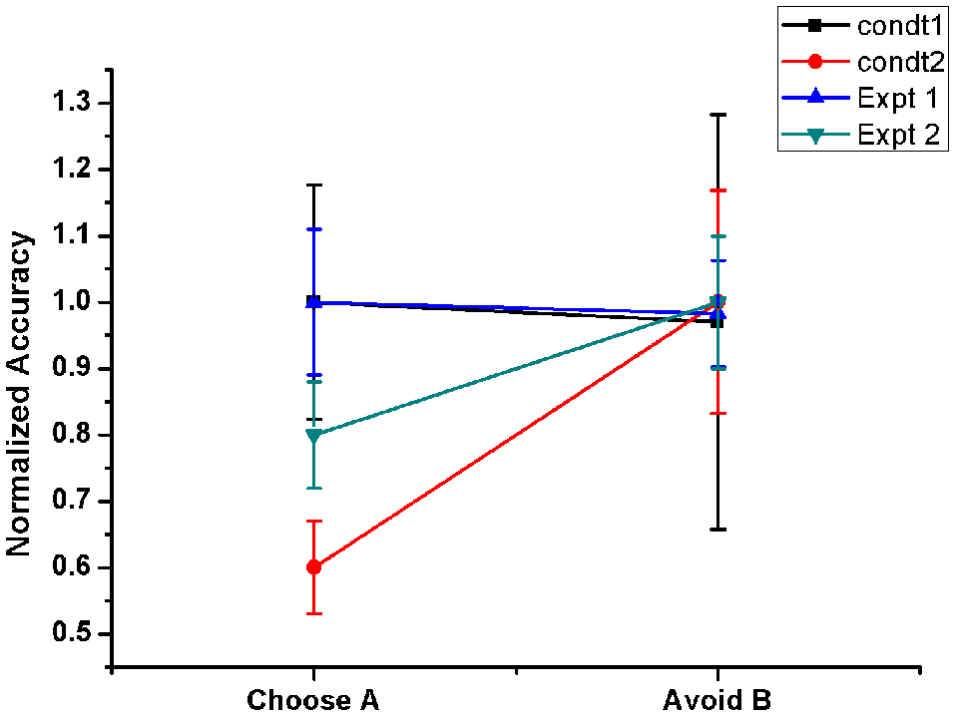
**Shows the Normalized performance of the model (with respect to maximum performance in each condition) in PD OFF state for two conditions**. Condt 1 and Condt 2 results are similar to the two contrasting behaviors of the subjects observed in Frank et al. (2007, Expt. 1) and Frank et al. (2004, Expt. 2) redrawn. Condt 1 showed no bias to punishment learning which was observed in Condt 2.

**Figure 7:**
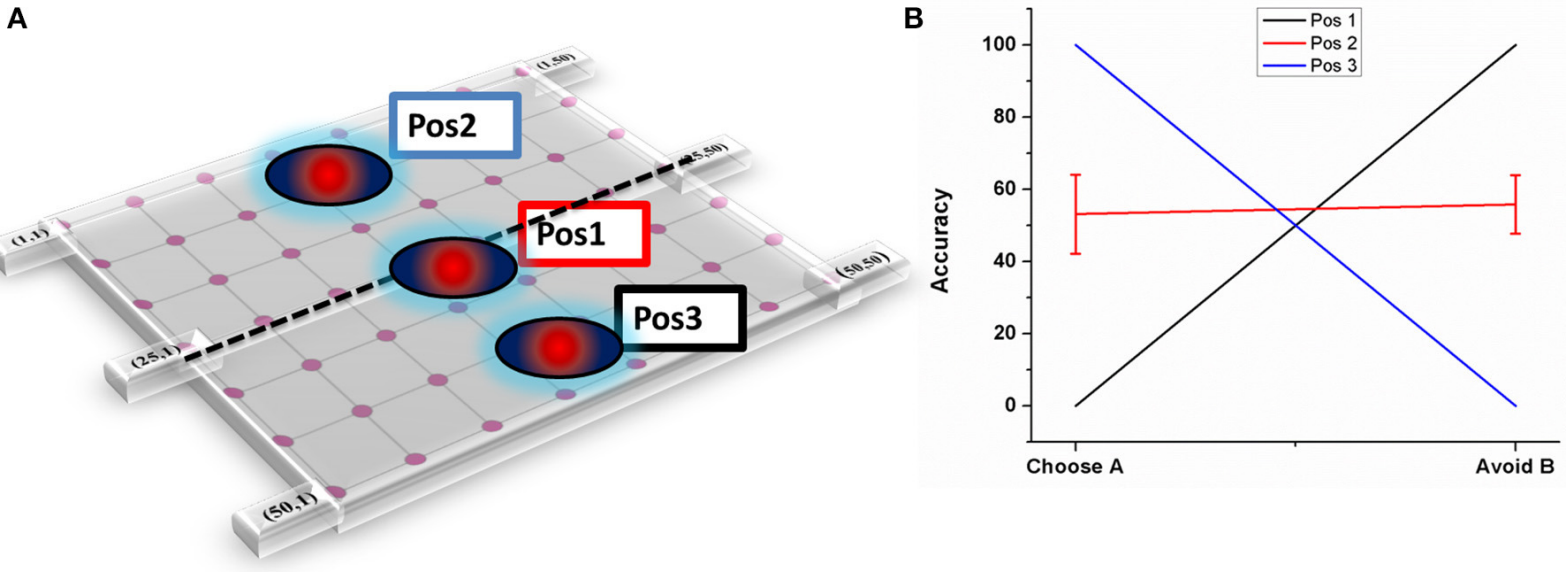
**Shows the effect of electrode position on performance (A) Graphical representation of the electrode position in STN lattice where Pos1 has the electrode center at the lattice point (25, 25), Pos 2 at lattice point (13, 13) and Pos 3 at (38, 38) (B) shows the performance of the model during stimulation of STN for each of the 3 positions**.

To this end, the model was presented with novel combinations of choices “A” and “B” similar to that mentioned in the earlier Section Choice/Avoidance Accuracy. The test was implemented on normals, PD OFF, PD ON (L-DOPA and DAA) (Figures 5, 6), and DBS conditions (Figure 7).

#### Normal and PD conditions

The results [normals, PD OFF, PD ON (L-DOPA)] obtained from the model were compared with that of from Frank et al. (2007). The results obtained from experiment (Figure 5A) and simulations (Figure 5B) were found to be similar. The mean accuracy (in terms of choosing “A” and avoiding “B”) with standard error (SE) obtained from the computational model in normal condition for choosing A (avoiding B) was 82.98 ± 2.68 (78.29 ± 5.08), PD OFF was 82.42 ± 2.03 (81.25 ± 7.39), PD ON (L-DOPA) was 73.29 ± 3.13 (51 ± 2.56), and PD ON-DAA was 56.1 ± 8 (57.5 ± 8.05). The mean performance calculated in selecting choice “A” among the four conditions were found to be significantly different at *p* = 0.05 level at [*F*_(4, 23)_ = 5.53, *P* = 0.003]. *Post-hoc* analysis using Bonferroni method showed a significant difference between PD ON-DAA condition and normals (*P* = 0.004) and PD OFF (*P* = 0.04). Similarly, a significant difference was observed among the 4 conditions for avoiding choice “B” at *P* = 0.05 level at [*F*_(4, 23)_ = 4.49, *P* = 0.01]. *Post-hoc* analysis using Bonferroni method showed a significant difference only between normals and PD ON (L-DOPA) condition (*P* = 0.03).

The PLT study conducted by Frank and group on 2 sets of PD OFF patient groups showed opposite results in terms of avoiding “B” choice. One group's performance was similar to normals (Frank et al., 2007) but the other patient pool showed a bias toward punishment learning (Frank et al., 2004), i.e., they learnt to avoid the punitive choice “B” better than to select the rewarding choice “A.” It was quite intriguing to observe that a similar patient pool exhibited two contrasting behaviors. In order to analyse this, we checked the effect of various model parameters on avoiding “B” performance. We observed that the lateral connectivity parameter within STN (*r*_*s*_) and GPe (*r*_*g*_) neurons played a critical role in altering the behavior. We used 2 set of values for lateral strengths of STN and GPe, i.e., [Condt1 = (*r*_*s*_ = 3.3) and (*r*_*g*_ = 0.7)], [Condt2 = (*r*_*s*_ = 1.43) and (*r*_*g*_ = 1.7)]. We observed that the performance obtained using Condt1 values was similar to patient behavior that showed no bias to reward and punishment whereas Condt2 values showed punishment biased behavior (Figure 6).

#### STN stimulation

Various experimental and clinical studies reported impulsivity in PD patients after stimulation of STN (Hershey et al., 2004; Smeding et al., 2006, 2009; Frank et al., 2007; Ballanger et al., 2009; Wylie et al., 2010) which was soon contradicted (Castrioto et al., 2015). Keeping this in mind we studied the effect of electrode position and antidromic activation on reward and punishment learning. Based on our earlier results that position of the electrode could be a potential factor for impulsivity in DBS subjects (Hershey et al., 2010; Mandali and Chakravarthy, 2015), we varied the position of the electrode and changed the percentage of antidromic activation keeping all other stimulation parameters constant.

##### Electrode position

As explained in Section Electrode position, three positions have been chosen (Figure 7A) and accuracy levels (in terms of choosing “A” and avoiding “B”) have been calculated. The mean accuracy levels for Pos 1 in choosing A (Avoiding B) was 0 (100), for Pos 2 it was 53.125 ± 10.72 (55.85 ± 8.8), and for Pos 3 it was 100(0). As it can be observed from the plot (Figure 7B), the model performance is biased to either reward-based (Pos 3) or punishment-based learning (Pos 1) based on the position of the electrode. As the final choice selection is dependent on GPi activity which is partly controlled by STN, the stimulation current's ability to vary the STN activity influenced the final action selection.

##### Antidromic activation effect

**Figure 8:**
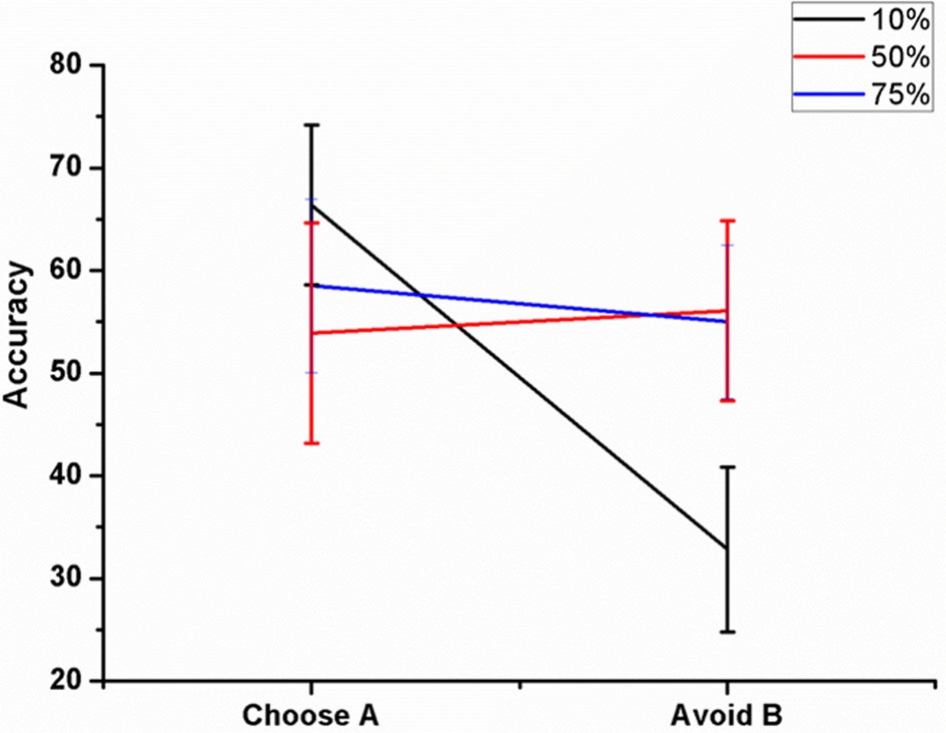
**Shows the effect of antidromic activation on the behavior during PLT on Accuracy obtained when the percentage of DBS current was varied (10, 50, and 75%)**.

We varied the percentage (10, 50, and 75%) of antidromic activation for a fixed position of electrode (Pos 2, Figure 7A), frequency (=130 Hz) and amplitude (200 pA) and observed the result in terms of the accuracy. The mean accuracy level in choosing A (avoiding B) for 10% was 66.4 ± 7.8 (32.81 ± 8.02), 50% was 53.9 ± 10.72 (56.09 ± 8.8), and for 75%, it was 58.5 ± 8.46 (55 ± 7.5) (Figure 8). We observed that for lower values of GPe activation, the model behaved similar to medication (L-DOPA), i.e., a bias toward reward learning but on further increase the model accuracy turned out to be similar for both reward and punishment learning.

The above results demonstrate the learning ability and performance (reward and punishment) under physiological and pathological condition. The simulation results suggest that change in STN dynamics, arising due to a change in STN lateral connection strength, seems to be a key contributing factor to altered behavior among PD patients. We also observed that stimulation parameters such as electrode position and antidromic activation are critical and influence reward and punishment learning.

### Reaction time and impulsivity

As explained in Section Reaction Time, we calculated the RT for each of the five conditions [Normals/PD OFF/PD ON (L-DOPA and DAA) and DBS] for correct and error trials.

#### Normal and PD conditions

**Figure 9:**
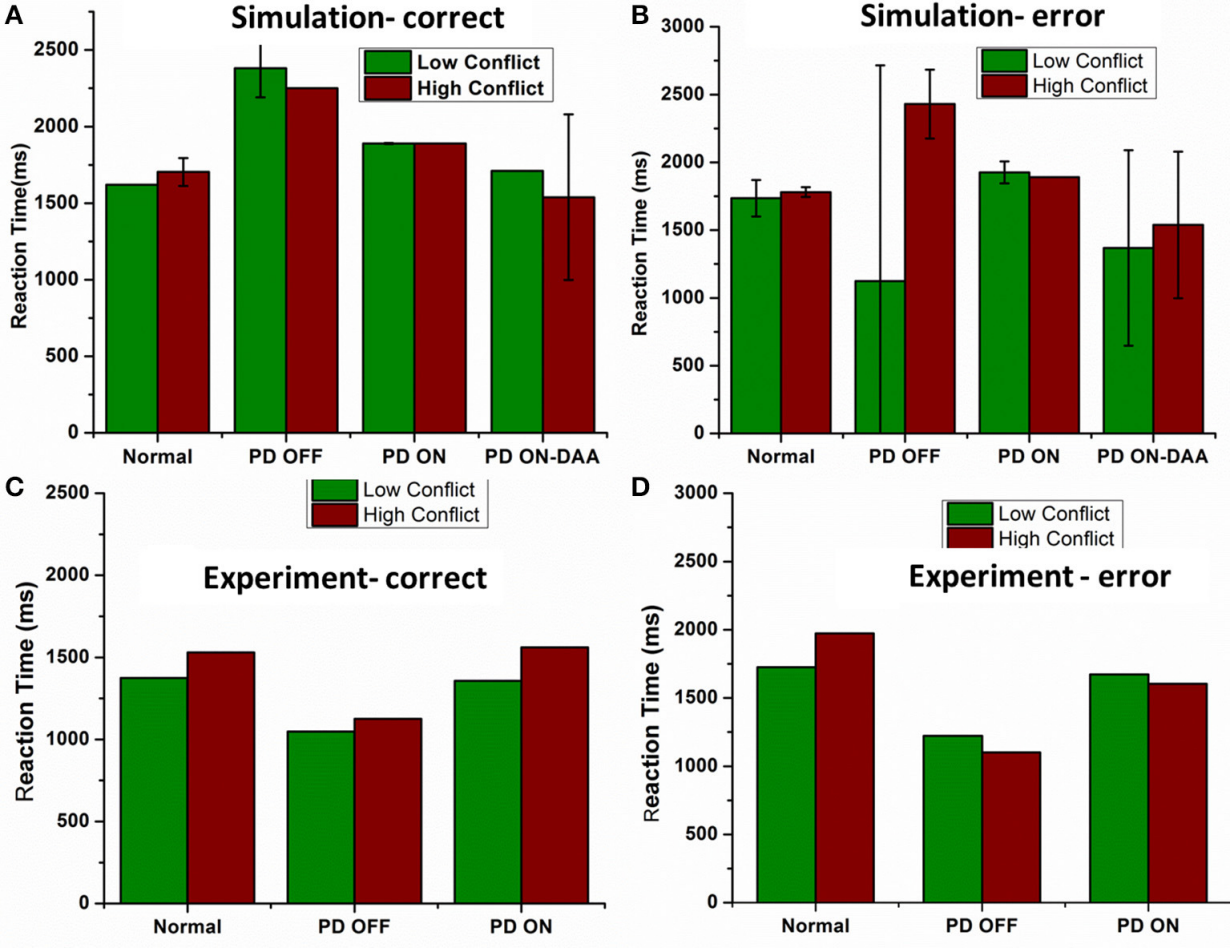
**Shows the reaction time in milliseconds (ms) for various conditions applied on the spiking BG model (A) Reaction time (ms) obtained from the model for all the four conditions Normals, PD OFF, PD ON (L-DOPA, DAA) for LC and HC condition in correct trials**. **(B)** Reaction time (ms) measured from the model for all the four conditions [Normals, PD OFF, PD ON (L-DOPA, DAA)] for LC and HC condition in error trials. Experimental Reaction time (ms) obtained from Normal, PD OFF and PD ON condition for **(C)** correct and **(D)** error trials from Frank et al. (2007).

The mean RT's (in milliseconds) with standard deviation obtained for correct trials in LC (HC) condition (Figure 9A) for normals is 1620 ± 0 (1703 ± 91.59); PD OFF is 2381.4 ± 190.91 (2250 ± 0); PD ON (L-DOPA) is 1890 ± 3.07 (1890 ± 0); and PD ON(DAA) is 1710 ± 0 (1539 ± 540.75). The RT for PD-DAA condition was the lowest for HC among all the other cases, suggesting impulsive behavior known to be present in dopamine agonist treated subjects (Voon et al., 2007). The mean RTs with SD obtained for error trials in LC (HC) condition (Figure 9B) for normals is 1735.7 ± 134.64 (1780 ± 35.4), PD OFF is 1125 ± 1591.1 (2430 ± 254.5), PD ON (L-DOPA) is 1926 ± 80.49 (1890 ± 0), and PD ON(DAA) is 1368 ± 720.9 (1539 ± 540.75). On performing ANOVA on reaction times in all the conditions, we observed a significant difference between correct (*P* < 0.0001) and error trials (*P* = 0.004).

Based on the theory that STN-GPe chaotic dynamics are responsible for the generation of noise that is crucial for exploration (Chakravarthy et al., 2010; Kalva et al., 2012; Chakravarthy, 2013; Mandali et al., 2015) and which is no longer produced in PD condition, we can safely assume the presence of bursting and synchronous activity in STN. This pathological bursting activity leads to two outcomes; (1) Increase in the firing rate of GPi neurons leading to longer RT and (2) Regularized bursting STN activity that lead to a deterministic activation of the GPi neurons without any noise eventually leading to non-variable reaction time (no standard deviation). Similar could be the case for PD ON condition where the bursting activity of STN in PD OFF condition modulated by medication is changed to a more regular spiking eventually leading to RT values with low variance. From Figure 9B of PD OFF condition, we can observe that model's RT is low for error trials. It is clear that due to faster/impulsive response the model performed poorly in those particular trials.

#### STN-DBS

We then checked for the effect of electrode position and antidromic activation on RT, as described earlier.

##### Electrode position

**Figure 10:**
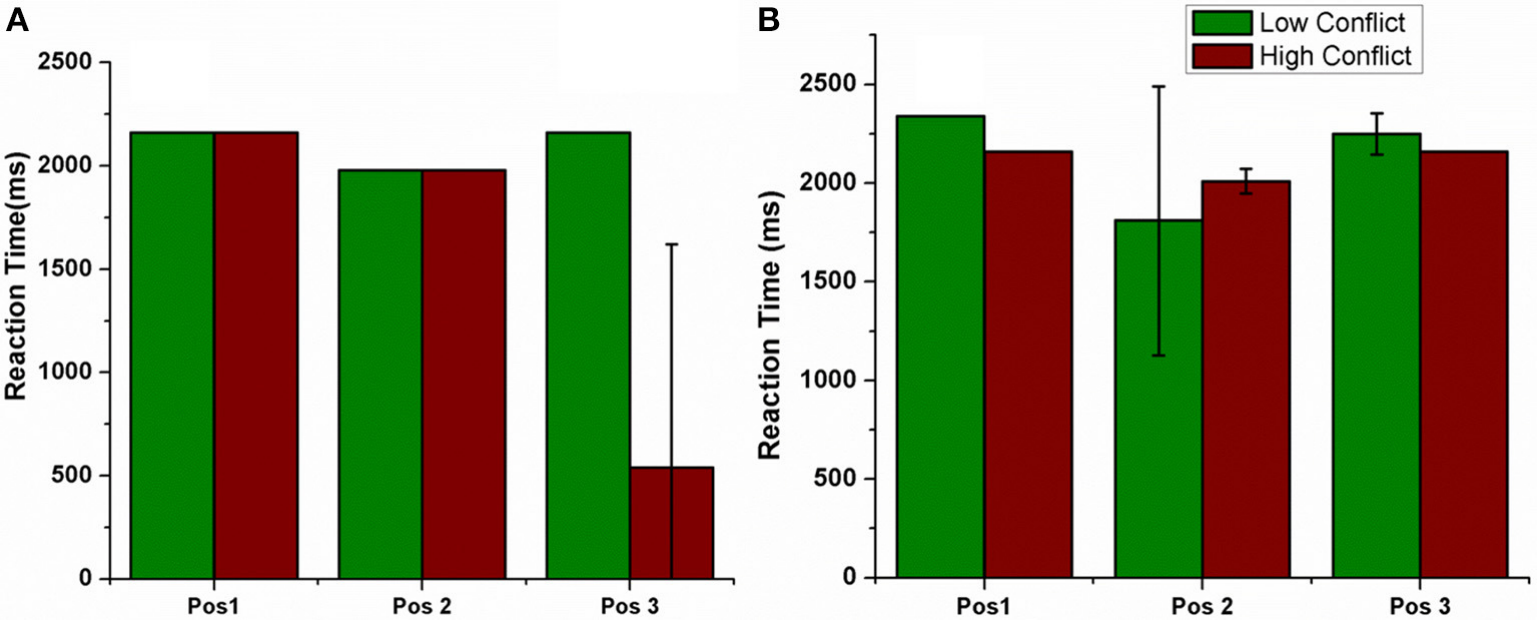
**Shows the RT for LC and HC conditions when the position of the electrode was changed**. Pos 1, Pos 2, Pos 3 are also described in Figure 7. **(A)** Correct, **(B)** Error trials.

The electrode was shifted between the three positions keeping all other stimulation parameters constant and the RT was measured in LC and HC trials (Figure 10). We observed that the RT decreased for HC condition, decreased for a specific electrode position (=Pos 3) (for both correct and error trials as plotted in Figure 10).

**Figure 11:**
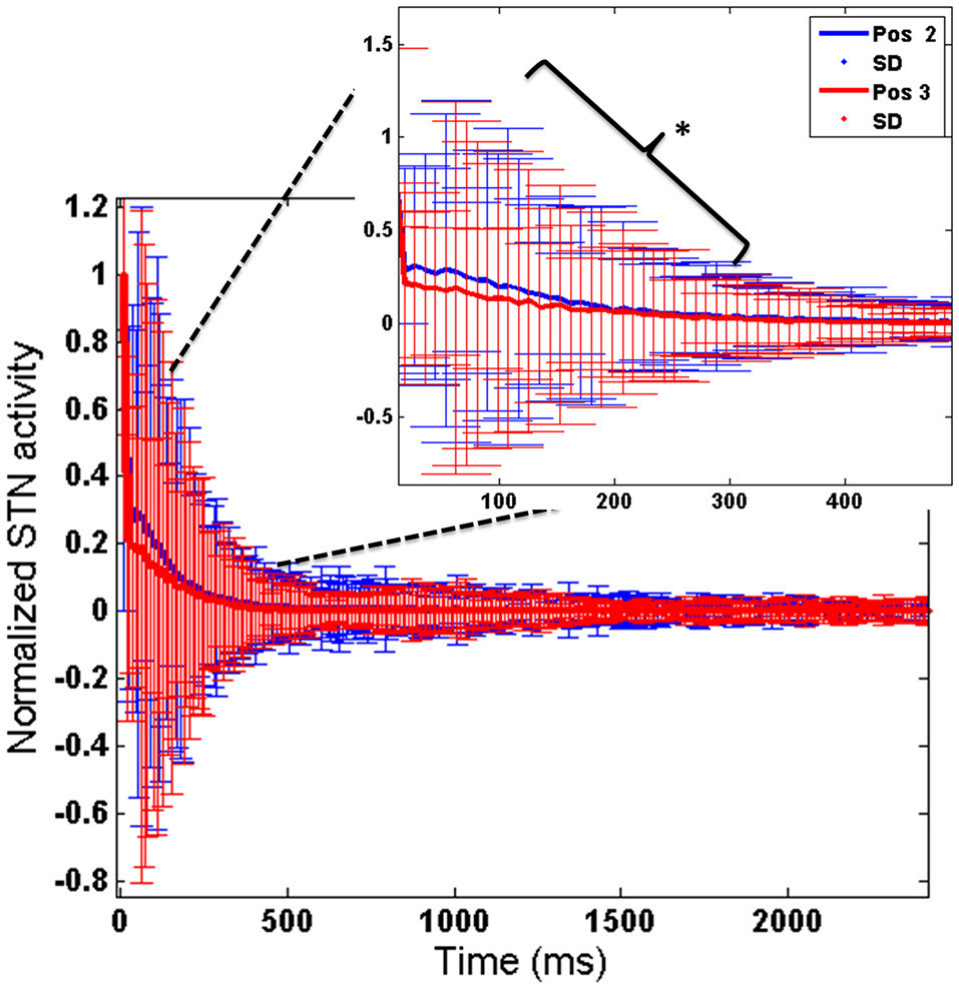
**Shows the STN activity for HC conditions for 2 electrode positions (Pos 2 and Pos 3)**. X-axis shows the simulation time and Y-axis shows the normalized STN activity with SD across time. The inset picture clearly shows a significant reduction in STN activity for Pos 3 of DBS electrode. ^*^Indicates the significance level at *p* < 0.05.

To further analyse the above obtained result (Figure 10), we observed the STN activity in HC conditions for the two electrode (Pos 2 and Pos 3) positions. This was to observe how the stimulation current affected the activity of STN neurons in HC condition. We observed that the STN activity for Pos 3 (which corresponds to decreased RT) was significantly lower (*t*-test at *p* = 0.05) than Pos 2 (Figure 11).

##### Antidromic activation effect on RT

**Figure 12:**
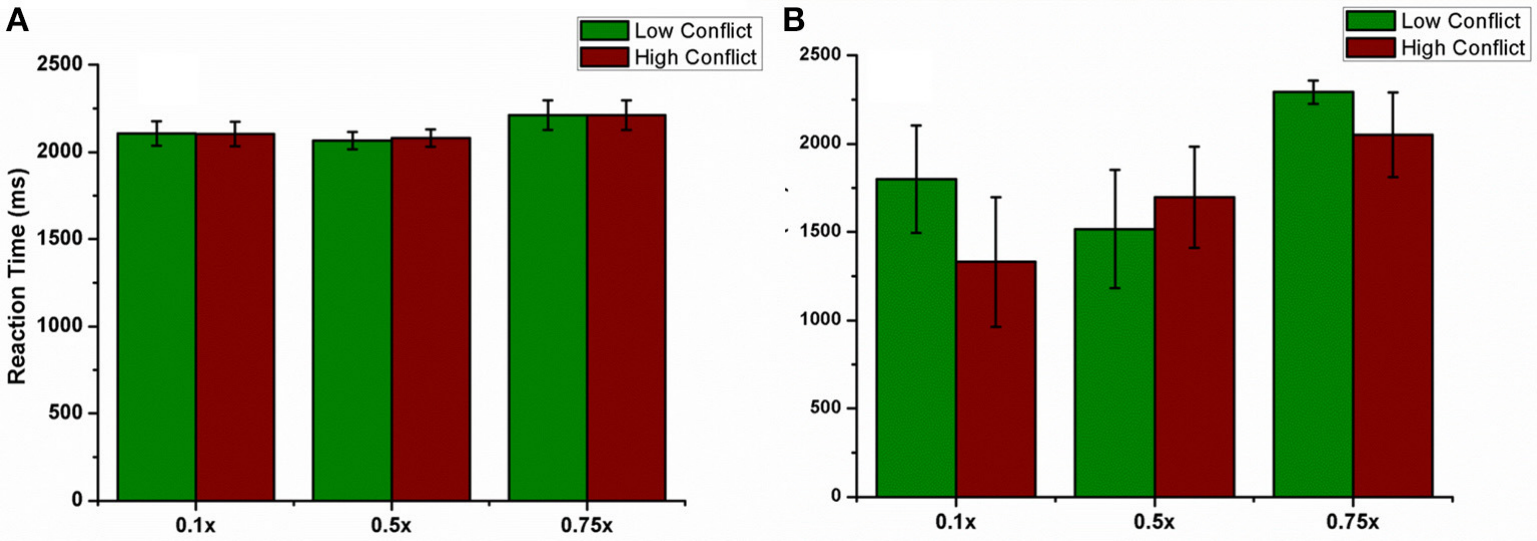
**shows the effect of antidromic activation on the behavior during PLT**. Reaction time obtained, **(A)** for correct trials and **(B)** for error trials when the percentage of DBS current was varied (10, 50, and 75%).

We also checked this antidromic effect on reaction time in both correct and error trials. We did not observe any difference in RT between LC and HC conditions in correct trials for any of the percentages. But RT's in error trials were low for HC trials for 10% and 75% of GPe activation. Only for 50% the model's RT for HC trial was higher than LC trial (Figures 12A,B). These results suggest a probable role of antidromic activation in controlling the STN activity.

This set of results study the impulsivity characteristics in various conditions of the model in terms of RT. The DBS electrode position not only modulates learning ability but also influences the RT by decreasing the STN activity in certain electrode positions. An increased activation of GPe neurons by DBS current also reduced the RT.

## Discussion

PD patients suffer not only from motor abnormalities, but also show signs of cognitive impairment in terms of working memory, learning and executive functions (Owen et al., 1992; Dubois and Pillon, 1996; Chaudhuri et al., 2006; Kehagia et al., 2010). Although therapeutic methods, such as medication as well as stimulation, relieve motor symptoms, they often cause side effects such as impulsivity, learning deficits (Frank et al., 2004, 2007; Voon et al., 2007). L-DOPA has been observed to interfere with learning and DAA are linked to impulse control disorders. Similarly, various experimental results drew attention to STN stimulation effects on cognitive aspects also (Hershey et al., 2004; Smeding et al., 2006; Temel et al., 2006; Frank et al., 2007). Experimental and modeling studies suggest the role of electrode parameters (position and current) on the behavioral outcome of the PD patients (Hershey et al., 2010; Mandali and Chakravarthy, 2015). We start the discussion by explaining the effect of medication and stimulation parameters on learning ability and then on impulsivity.

### Learning

We first studied the learning and performance aspects in normals, PD OFF, PD ON (L-DOPA and DAA), and DBS conditions. The learning curve for each of the rewarding choices (A/C/E) obtained from the model (Figure 3) in PD OFF condition was compared with the results from Zaghloul et al. (2012). By the end of training the model's training accuracy in PD OFF reached the actual reward probabilities of the choices, confirming the learning ability of the spiking model. In normals and PD ON (L-DOPA) cases, the model was able to reach the expected reward probability value, which did not occur in PD ON (DAA) and DBS conditions.

### Testing accuracy and choice/avoidance accuracy

The model's ability to differentiate between a high rewarding and low rewarding choice in each of the physiological and pathological conditions [PD OFF, PD medicated conditions (L-DOPA and DAA), and stimulation] was tested by comparing DRE and testing accuracy.

#### Normal and PD conditions

The results from PD OFF (Figure 4B) showed a linear relationship between DRE and testing accuracy which indicated the model's ability to choose a particular choice with high DRE and avoid it otherwise. Another way of explaining the same result is by analysing the performance in terms of Choice/Avoidance accuracy, where the accuracy levels were not significantly different for both choosing A and avoiding B cases. This trend in Normals and PD OFF conditions can also be noticed in Figures 5A,B. But this behavior was absent in both of the medicated conditions (L-DOPA and DAA). Even for stimuli where one of the choices was punitive in nature, the testing accuracies (i.e., selecting the particular choice) obtained after training were high (Figures 4C,D). This suggests that the model in PD ON (L-DOPA) condition could not learn from punishments and continued to select the lower rewarding choice. This can be further verified from the *post-hoc* analysis where only the accuracy of PD ON (L-DOPA) was significantly different from all other conditions in avoiding “B.” This behavior was also experimentally observed where PD patients under medication tend to learn more from rewards than punishments (Frank et al., 2004, 2007). This can be accounted by the medication term (δ_med_ = 2) (Equation 5) which prevents the model to learn from punishments. The higher amounts of DA due to medication prevented the dip even on the selection of punitive choices. The model's performance in DAA condition did not yield good accuracy in reward learning but performed better than L-DOPA condition in punishment learning (Figure 5). This could be observed in the DRE vs. accuracy plot (Figure 4D) where the testing accuracy in DAA condition was low for DRE values (< 0) when compared to that obtained in L-DOPA (Figure 4C) condition.

To address how two groups of PD OFF subjects can show contrasting behavior in punishment learning (Frank et al., 2004, 2007), we modified the connection parameters within STN and GPe in the model and studied the performance (Figure 6). Our simulation results show that the lateral connection strength and the level of synchrony in STN and GPe neurons can influence the behavior. Earlier studies by Rubchinsky and colleagues suggested the presence of intermittent synchrony in PD patients (Park et al., 2011). The final action selection at the level of GPi was influenced by the STN-GPe oscillations through indirect pathway. Therefore, a difference in lateral connection radius within these excitatory-inhibitory neurons led to subtle changes in their synchrony level which eventually reflected at the level of decision making.

#### STN-DBS

Another important aspect of our study is to observe if there is any effect of stimulation parameters such as electrode position and increased antidromic activation of DBS on learning. As the position of electrode was changed (Figure 7) the model switched from reward-based to punishment-based learning. The presence of parallel BG loops with different functions has been well known anatomically (Alexander et al., 1986); the points of intersection of these loops with STN have also been topographically mapped (Hamani et al., 2004). It is possible that variation in the electrode position physiologically could be related to an activation of different areas which are known to modulate reward and punishment learning differently (Wächter et al., 2009). For a specific position (Figure 7- Pos 2), the accuracy level for choosing A and avoiding B was same but reduced compared to normals. These results show that electrode might be playing an important role in the cognitive function of the subject. Apart from electrode position, we also studied the effect of antidromic activation of GPe neurons due to stimulation in STN neurons (Figure 8). For 10% of the stimulating current affecting the GPe neurons, the behavior in terms of accuracy was quite similar to PD-ON (L-DOPA) results. For higher percentages (i.e., 50 and 75%), the behavior was similar to experimental DBS results.

### Reaction time and impulsivity

Based on the experimental evidence that an increase in STN activity was observed during HC conditions, we analyzed the reaction time for each of the conditions in LC and HC cases in each of the five conditions.

#### Normal and PD conditions

We observed that the model in normal condition took more time to make a choice during HC case compared to that in LC in both correct and error trials (Figures 9A,B). The impulsivity behavior observed clinically due to DAA medication (Voon et al., 2007; Ondo and Lai, 2008) was captured by the model where we observed a lower RT for HC case. PD-ON DAA condition showed the lowest RT compared to other conditions and PD-OFF the highest as shown in Frank et al. (2007) and Hauptmann and Tass (2007). High STN activity in untreated PD condition could make the model take longer to reach the threshold thus leading to a higher reaction time. DAAs which selectively affect D2 receptors could decrease the STN activity making the system to respond faster leading to incorrect and impulsive decisions. The model in PD OFF condition also showed an increase in RT for HC case during error trials but a decrease during correct ones.

#### STN-DBS

The concept of impulsivity due to DBS was studied by varying parameters such as electrode contact position within STN nucleus and inducing antidromic activation of GPe neurons. We observed that the RTs were different for different electrode positions and a lower RT was obtained for HC case during both correct and error trials for a specific electrode position (Pos 3). To further analyse why such behavior was observed, we checked the STN activity for each of the positions (Pos 2, Pos 3) in HC conditions (Figure 10). We observed a significant (*P* < 0.05) decrease in STN activity in Pos 3 condition compared to Pos 2 during the first 600 ms of stimulation (Figure 11). We hypothesize that it is due to this initial difference in the STN activity that a reduction in RT was observed.

Based on the theory which considers the possibility for antidromic activation of GPe neurons during STN stimulation (Hauptmann and Tass, 2007; Montgomery and Gale, 2008), we studied the RT for various levels of GPe neuronal activation (Figures 12A,B). For a fixed position of DBS electrode at the lattice position (25, 25), there was no significant difference in the RT for correct trials. But for error trials, the percentage of GPe activation affected the RT especially in HC case. Only for 50% of the cases, the RT in HC case was higher than that obtained in LC. A higher activation of GPe neurons (=75%) though shows normal learning behavior gives a decreased RT in error trials as observed in experiment (Frank et al., 2007). These results show that higher antidromic activation of GPe neurons could be a probable reason for the observed impulsivity in DBS patients.

We also studied the effect of the inhibitory connection from GPe to GPi on accuracy and RT in normal. Preliminary results from the model showed no significant difference in accuracy or RT when simulated with and without this connection (results not included). But further analysis has to be performed to fully understand the functional significance of this anatomical connection.

### Conclusions, limitations, and future work

The symptom profiles for various patient types are diverse and so are their responses to either medication and/or stimulation. Our spiking BG model gives an insight into the patient's response to each of these therapies (learning and RT) which might help to suggest alternative protocols. We also emphasize the importance of synchrony in STN critically modulated by the lateral connections within STN, and how STN influences the final behavior in patients, which is not accounted by many other computational BG models.

In future, we would like to address our model limitations in terms of explaining the lower RT observed in PD OFF patients. We believe that incorporating risk based approach (Balasubramani et al., 2015) in to our current model would capture the result of lower RT in PD OFF condition. Also, we would like to include the inhibitory GPe→GPi connection and hyper direct pathway connection in to the network. Another limitation which we would like to address is the spatial definition and boundaries in STN based on the functional cortico-BG loops. We are yet to fully understand the physiological effects of DBS on STN in terms of behavior and different frequency bands and would expand the model with more realistic connectivity and integration of other BG nuclei.

## Author contributions

AM developed the computational model, simulated the tasks, analyzed the results, and prepared the manuscript. VSC developed the computational model, simulated the tasks, analyzed the results, and prepared the manuscript.

### Conflict of interest statement

The authors declare that the research was conducted in the absence of any commercial or financial relationships that could be construed as a potential conflict of interest.
