# Supplementary material for: Probing the Role of Medication, DBS Electrode Position, and Antidromic Activation on Impulsivity Using a Computational Model of Basal Ganglia
Source: Front Hum Neurosci. 2016 Sep 12;10:450. doi: 10.3389/fnhum.2016.00450 (PMC5019076; doi:10.3389/fnhum.2016.00450)
Supplement: Supplementary file 1 [file DataSheet1.DOCX]

**Synaptic Connections in spiking BG model**

The synaptic connectivity between the nuclei was considered as one to one as in ([Dovzhenok and Rubchinsky, 2012](#_ENREF_25)) and was modeled as (similar to ([Humphries et al., 2009](#_ENREF_50)))

$\tau_{\mathrm{Re}cep}*\frac{dh_{ij}^{x\to y}}{dt}=-h_{ij}^{x\to y}(t)+S_{ij}^{x}(t)$ (1)

$I_{ij}^{x\to y}(t)=W_{x\to y}*h_{ij}^{x\to y}(t)*(E_{\mathrm{Re}cep}-V_{ij}^{y}(t))$ (2)

The effect of voltage-dependent magnesium channel on NMDA current ([Jahr and Stevens, 1990](#_ENREF_55)) was modeled as,

$B_{ij}(v)=\frac{1}{1+(\frac{Mg^{2+}}{3.57}*e^{-0.062*V_{ij}^{y}(t)})}$ (3)

Where τ_Recep_= decay constant for synaptic receptor, E_Recep_=receptor associated synaptic potential (Recep=AMPA/GABA/NMDA), S^x^_ij_=Spiking activity of neuron ‘x’ at time‘t’, h_ij_^x→y^= gating variablel for the synaptic current from ‘x’ to ‘y’,W_x→y_= synaptic weight from neuron ‘x’ to ‘y’, Mg^2+^ =Magnesium ion concentration and V^y^_ij_= membrane potential of the neuron ‘y’. The time constants of GABA, AMPA and NMDA in STN and GPe were chosen from ([Götz et al., 1997](#_ENREF_37)) are given in Table 1. All the synaptic connections with their respective variables are described in Table 2 and the values of parameters are given in Table 1.

**Lateral Connections in STN and GPe neurons**

$R_{s}=\frac{r_{s}}{(cD21*DA)};R_{g}=\frac{r_{g}}{(1-cD21*DA)};w_{ij,pq}^{m\to m}=A_{m}*e^{\frac{-d^{2}_{ij,pq}}{R_{m}^{2}}},d_{ij,pq}^{2}=(i-p)^{2}+(j-q)^{2}$ (4)

$I_{ij}^{\mathrm{Re}ceplat}=B_{ij}\left(v\right)*\sum_{p=1}^{n} \sum_{q=1}^{n} w_{ij,pq}^{x\to x}*h_{ij}^{\mathrm{Re}cep\to x}(t)*(E_{\mathrm{Re}cep}-V_{ij}^{x}(t))$ (5)

where r_s_ = constant variance of STN Gaussian, r_g_= constant variance of GPe Gaussian, R_s_ = changed variance of STN Gaussian due to the effect of dopamine, R_g_= changed variance of GPe Gaussian due to the effect of dopamine, cD21= a constant that determines the effect of DA on STN and GPe laterals, w_ij_^m→m^=lateral weight matrix of neuron‘m’at location (i, j), d_ij, pq_= distance from center neuron (p,q), R_m_=R_g_ (or) R_s_, A_m_=strength of lateral synapse, m =STN or GPe neuron. All parameter values are given in Table A.I.

$W_{x\to y}=\left(1-cd2*DA\right)*w_{x\to y}$ (6)

where the synapses are GPe→STN and STN→GPe. A similar method for DA-dependent synaptic modulation on striatal neurons was also used in ([Humphries et al., 2009](#_ENREF_50)).

Table A.I gives the values and the description of the parameters used in the model and simulation.

| **Parameter** | **Values with description** | | | |
| --- | --- | --- | --- | --- |
|  | STN | | GPe | GPi |
| Izhikevich parameters | a=0.005,b=0.265,c=-65, d=1.5 | | a=0.1,b=0.2,c=-65, d=2 | a=0.1, b=0.2, c=-65, d=2 |
| External current (I) | I_STN_=30 | | I_GPe_=10 | I_GPi_=10 |
| W_StrD1→GPi_ | 0.8 | | Synaptic weight between D1 striatum and GPi | |
| W_StrD2→GPe_ | 1 | | Synaptic weight between D2 striatum and GPe | |
| DA | 0.1 to 0.9 in increments of 0.1 | | | |
| A_D1_ | 10 | Amplitude of GABAergic current from D1 striatum to GPi neurons due to DA | | |
| A_D2_ | 7.5 | Amplitude of GABAergic current from D2 striatum to GPe neurons due to DA | | |
| λ^Str^ | 7.5 | Slope of the Gain functions (cD1 and cD2) | | |
| Mg^2+^ | 1nM | Concentration of Magnesium ions in nM | | |
| E_AMPA_ | 0mv | Synaptic potential of AMPA receptor-associated channel | | |
| E_NMDA_ | 0mV | Synaptic potential of NMDA receptor-associated channel | | |
| E_GABA_ | -60mV | Synaptic potential of GABA receptor-associated channel | | |
| w_sg_ | 1 | Synaptic weight for excitatory STN to GPe projection | | |
| w_gs_ | 20 | Synaptic weight for inhibitory GPe to STN projection | | |
| cd2 | 0.1 | Parameter that affects the STN→GPe (w_sg_) and GPe→STN(w_gs_) weights | | |
| τ_AMPA_ | 6ms | Time decay constant for AMPA receptor | | |
| τ_NMDA_ | 160ms | Time decay constant for NMDA receptor | | |
| τ_GABA_ | 4ms | Time decay constant for GABA receptor | | |
| τ_NMDA_GPi_ | 67ms | Time decay constant for NMDA receptor of GPi neurons | | |
| r_s_ | 1 | Radius of STN laterals Gaussian | | |
| r_g_ | 0.5 | Radius of GPe laterals Gaussian | | |
| cD21 | 0.1 | Parameter that affects the radius of STN and GPe laterals | | |
| A_GPe_ | 1 | Synaptic strength within GPe laterals | | |
| A_STN_ | 0.2 | Synaptic strength within STN laterals | | |
| nlat_STN__ | 5 | # of lateral connections considered in STN neurons | | |
| nlat_GPe__ | 11 | # of lateral connections considered in GPe neurons | | |
| w_STN→GPi_ | 1.15 | Synaptic weight between STN and GPi | | |

^Note: STN= Sub Thalamic Nucleus, GPe= Globus Pallidus Externa, GPi= Globus Pallidus Interna^

**Total Synaptic currents received by each neuron**

Total synaptic currents received by GPe neurons

The total synaptic current received by a GPe neuron at lattice position (i,j) is the summation of GABAergic input from the D2-expressing striatal MSNs ([Gerfen et al., 1990](#_ENREF_33)) (eqn. 5), glutamatergic current from STN considering both AMPA and NMDA currents ([Götz et al., 1997](#_ENREF_37)) (eqn. 5) and the inhibitory lateral current form other GPe neurons (eqn. 8). The influence of DA on the GABAergic current from D2 striatum to GPe neuron ([Hadipour-Niktarash et al., 2012](#_ENREF_42)) was accounted by the variable cD2.

$I_{ij}^{GPesyn}=I_{ij}^{GABAlat}+I_{ij}^{NMDA\to GPe}+I_{ij}^{AMPA\to GPe}+ I_{ij}^{StrD2\to GPe}*cD2$ (7)

$cD2=\frac{A_{D2}}{1+\exp(\lambda^{Str}*DA)}$ (8)

Where I_ij_^GABAlat^ = the inhibitory lateral GABAergic current from other GPe neurons, I_ij_^NMDA→GPe^ = excitatory glutamatergic current from STN neuron due to NMDA receptor, I_ij_^AMPA→GPe^ = excitatory glutamatergic current from STN neuron due to AMPA receptor, I_ij_^StrD2→GPe^ = inhibitory GABAergic current from D2 striatum, cD2= Gain parameter that affects the GABAergic D2 striatal current.

Total synaptic currents received by STN neurons

The total synaptic current received by an STN neuron at lattice position (i,j) is summation of GABAergic current from GPe neurons([Fan et al., 2012](#_ENREF_27)) (eqn. 5) and glutamatergic input (both AMPA and NMDA) from other STN ([Kita et al., 1983](#_ENREF_58)) eqn.(8).

$I_{ij}^{STNsyn}=I_{ij}^{GABA\to STN}+I_{ij}^{NMDAlat}+I_{ij}^{AMPAlat}$ (9)

Where I_ij_^NMDAlat^ = excitatory glutamatergic current from collateral STN neurons due to NMDA receptor, I_ij_^AMPAlat^ = excitatory glutamatergic current from collateral STN neurons due to AMPA receptor,I_ij_^GABA→STN^ = inhibitory GABAergic current from GPe neuron.

Total synaptic currents received by GPi neurons

The total synaptic current received by a GPi neuron at lattice position (i,j) is a summation of GABAergic currents from D1 striatal neurons ([Gerfen et al., 1990](#_ENREF_33))and glutamatergic (both AMPA and NMDA) input from STN neurons ([Gerfen and Surmeier, 2011](#_ENREF_34)). The increase in GABAergic current from D1 striatum to GPi neurons due to DA modulation ([Kliem et al., 2007](#_ENREF_60)) was taken into account by the variable cD1.

$I_{ij}^{GPisyn}=I_{ij}^{NMDA\to GPi}+I_{ij}^{AMPA\to GPi}+I_{ij}^{StrD1\to GPi}*cD1$ (10)

$cD1=\frac{A_{D1}}{1+\exp(-\lambda^{Str}*(DA-1))}$ (11)

Where I_ij_^NMDA→GPi^ = excitatory glutamatergic current from STN neuron due to NMDA receptor, I_ij_^AMPA→GPi^ = excitatory glutamatergic current from STN neuron due to AMPA receptor, I_ij_^StrD1→GPi^ = inhibitory GABAergic current from D1 striatum, cD1= Gain parameter that affects the GABAergic striatal current.

Table A.II gives a description of all the synaptic variables of various synaptic currents modeled using eqns. (4, 8) in Section 2.2.1

| **Variable** | **Description** |
| --- | --- |
| $h_{ij}^{StrD1x\to GPi }$ | Gating variables for GPi neuron due to GABAergic projections from D1 striatum. ‘x’ represents the input #. For example, if there are 2 inputs presented to the model, x=1, 2. |
| $h_{ij}^{StrD2x\to GPe }$ | Gating variable for GPe neuron due to GABAergic projections from D2 striatum |
| $h_{ij}^{AMPA\to GPe}/h_{ij}^{NMDA\to GPe}$ | Gating variable for GPe neuron due to glutamatergic input from STN due to either NMDA or AMPA receptor. |
| $h_{ij}^{GABA\to STN}$ | Gating variable for STN neuron due to GABAergic input from GPe neuron |
| $h_{ij}^{AMPA\to STN}/h_{ij}^{NMDA\to STN}$ | Gating variable for STN neuron due to glutamatergic input from its collaterals due to either NMDA or AMPA receptor. |
| $h_{ij}^{GABA\to GPe}$ | Gating variable for GPe neuron due to GABAergic input from its collaterals. |
| $I_{ij}^{StrD1\to GPi}$ | Inhibitory GABAergic current to GPi neuron from D1 striatum |
| $I_{ij}^{StrD2\to GPe}$ | Inhibitory GABAergic current to GPe neuron from D2 striatum |
| $I_{ij}^{AMPA\to GPe}/I_{ij}^{NMDA\to GPe}$ | Excitatory glutamatergic current (AMPA/NMDA) from STN neuron to GPe neuron |
| $I_{ij}^{GABA\to STN}$ | Inhibitory GABAergic current from GPe neuron to STN neuron |
| $I_{ij}^{AMPAlat}/I_{ij}^{NMDAlat}$ | Excitatory glutamatergic current (AMPA/NMDA) from STN neuron to STN neuron due to collateral synapses. |
| $I_{ij}^{GABAlat}$ | Inhibitory GABAergic current from GPe neuron to GPe neuron due to collateral synapses. |
| $S_{ij}^{GPe}$ | Spiking activity of GPe neuron at location (i,j) at time ‘t’. |
| $S_{ij}^{STN}$ | Spiking activity of STN neuron at location (i,j) at time ‘t’. |
| $S_{ij}^{Dxy}$ | Spiking activity of striatum at location (i,j) at time ‘t’. The variable ‘y’ represents either D1 striatum (=1) or D2 striatum (=2) for an input stimulus ‘x’. |

^Note: STN= Sub Thalamic Nucleus, GPe= Globus Pallidus Externa, GPi= Globus Pallidus Interna^
